# In vivo optical cellular diagnosis for uterine cervical or vaginal intraepithelial neoplasia using flexible gastrointestinal endocytoscopy -a prospective pilot study-

**DOI:** 10.1186/s12885-020-07460-6

**Published:** 2020-10-02

**Authors:** Shoko Ono, Ayako Nozaki, Kana Matsuda, Emi Takakuwa, Naoya Sakamoto, Hidemichi Watari

**Affiliations:** 1 Department of Gastroenterology, Hokkaido University Hospital, Nishi-4, Kita-15, Kita-ku, Sapporo, 060-8648 Japan; 2 Department of Obstetrics and Gynecology, Hokkaido University Hospital, Nishi-4, Kita-15, Kita-ku, Sapporo, 060-8648 Japan; 3 Department of Gastroenterology and Hepatology, Faculty of Medicine and Graduate School of Medicine, Hokkaido University, Nishi-7, Kita-15, Kita-ku, Sapporo, 060-8638 Japan; 4 Department of Surgical Pathology, Hokkaido University Hospital, Nishi-4, Kita-15, Kita-ku, Sapporo, 060-8648 Japan

**Keywords:** Cervical intraepithelial Neoplasia, Colposcopy, Vaginal neoplasms, Endoscopes, ECA classification

## Abstract

**Backgrouund:**

For patients with any kind of atypical squamous intraepithelial lesion of the uterine cervix or vagina, colposcopy and punch biopsy are common procedures for histological determination following cytology. However, colposcopy-guided biopsy does not provide a high level of diagnostic accuracy. The aim of this study was to determine the usefulness of optical biopsy in vivo using endocytoscopy compared with conventional procedures using colposcopy.

**Methods:**

Between May 2018 and March 2019, patients who were scheduled for cervical conization or mapping biopsies of the vagina *were* prospectively enrolled. Endocytoscopy was performed by senior endoscopists prior to scheduled procedures, and endocytoscopic images and biopsy samples were taken from the most prominent site and surrounding area of the cervical or vaginal lesions. The collection process of images was randomized and anonymous, and three doctors separately evaluated the images according to the ECA classification. ECA 4 and 5 are indicative of endoscopic malignancy. The primary endpoint was diagnostic accuracy (benign or malignant: cervical intraepithelial neoplasia (CIN) 3 or vaginal intraepithelial neoplasia (VAIN) 3 or worse) of cell images at the most prominent site in each patient.

**Results:**

A total of 28 consecutive patients were enrolled. Sensitivity, specificity, positive predictive value, negative predictive value and accuracy of endocytoscopic images were 95.0% (84.8–98.6%), 87.5% (61.9–96.5%), 95.0% (84.8–98.6%), 87.5% (61.9–96.5%) and 92.9% (78.2–98.0%), respectively. Inter-observer agreement among three reviewers was 0.78 (0.08–9.88, *P* < 0.01). On the other hand, the accuracy of colposcopy-guided biopsy was 74.1% (64.0–84.0%).

**Conclusions:**

Optical cell diagnosis of cervical or vaginal intraepithelial neoplasia using endocytoscopy provides a high level of diagnostic accuracy.

**Trial registration:**

The study was registered with the UMIN database (ID: 000031712).

UMIN000031712*.* Registered 16 March 2017*,*

## Background

Cervical cancer is the 4th-most commonly diagnosed cancer (6.6% of total cases) and the 4th-leading cause of cancer death (7.5% of total cancer deaths) in women worldwide [1]. The estimated number of new cases of cervical cancer worldwide every year is 527,600 and the estimated number of deaths from cervical cancer every year is 265,700 [1]. Generally, screening for cervical cancer is performed by cytology (Papanicolaou test) using cervical smears. According to the categories of the 2001 Bethesda system, patients with any kind of atypical squamous intraepithelial lesion of the uterine cervix or vagina are recommended by the American Society for Colposcopy and Cervical Pathology to receive colposcopy or an immediate loop electrosurgical excision procedure [2, 3]. Colposcopy and punch biopsy are common procedures for histological determination following cytology. The 2011 Japan Society of Gynecologic Oncology guidelines recommend cervical conization for patients with cervical intraepithelial neoplasia (CIN) 3 or worse lesions diagnosed by biopsy [4, 5]. However, colposcopy-guided biopsy does not always provide high diagnostic accuracy [6, 7].

Recently, endocytoscopy, which provides in vivo cellular imaging for gastrointestinal lesions, has been developed and instruments have become commercially available in Japan since 2017. The most advanced endocytoscope is a flexible scope for the digestive tract that enables conventional observation, magnifying observation (× 100) and microscopic visualization (× 500) by using a hand lever [8]. There have been some reports about the usefulness of endocytoscopy for diagnosis of gastrointestinal intraepithelial neoplasia and non-epithelial neoplasia [9–13]. The aim of this study was to determine whether optical biopsy in vivo using endocytoscopy has better diagnostic performance than that of conventional procedures using colposcopy.

## Methods

### Subjects

Between May 2018 and March 2019, patients who were scheduled for cervical conization or mapping biopsies under *lumbar anesthesia* were prospectively enrolled. The inclusion criterion was indication for treatment of CIN or vaginal intraepithelial neoplasia (VAIN) detected by colposcopy and/or cytology. Patients who were allergic to methylene blue, patients who were under 20 years old and patients for whom endocytoscopy would adversely affect the scheduled operation were excluded. Written informed consent was obtained from all patients before enrolment, and this study was performed in accordance with the ethical standards detailed in the Declaration of Helsinki. The authors’ institutional ethics committee approved this study on March 15, 2017 (Hokkaido University Hospital Review Board 017–0296). The study was registered with the UMIN database (ID: 000031712) on March 16, 2017.

## Methods

Supplemental Fig. 1 shows the flow of our study.

### Endocytoscopy

Patients under lumbar spinal anesthesia and sedation were placed in the lithotomy position in the operating room. After inserting a Cusco speculum into the vagina, vaginal irrigation was performed. Cell imaging in vivo was performed using endocytoscopy (H290EC) with a video processor (CV290) and light source (CLV290; Olympus Medical Systems Corp., Tokyo, Japan). This equipment provides about 500 magnification and an observation range of 570  ×  500 μm with an outer diameter of 9.7 mm [9]. The endoscope was inserted into the vagina by senior endoscopists (S. O. and K. M.) and surfaces of the cervical or vaginal lesions were observed. After washing carefully, the cells were stained using 0.1% methylene blue and endocytoscopic images of malignant areas and surrounding areas (two areas of each) were obtained. An additional movie file shows this in more detail [see Additional file 1]. Target biopsies through the endoscope were performed from each area (Radial Jaw™ 4 biopsy forceps, Boston Scientific Corporation, USA). During the procedure, we took about 5 min. After completing the endocytoscopy, scheduled procedures were performed by gynecologists.


**Additional file 1.** The procedure of endocytoscopy for cervix. After staining using 0.1% methylene blue, malignant area is focuses by endocytoscopy. Target biopsy is obtained, and surrounding area is focused. ECS 5 (increase of the ratio of nucleus ± cytoplasm and irregular arrangement of enlarged and blurred nuclei) in malignant area and ECS 3 (Increase of cells without enlarged nuclei) in surrounding area are observed.

### Review of endocytoscopic images

**Fig. 1:**
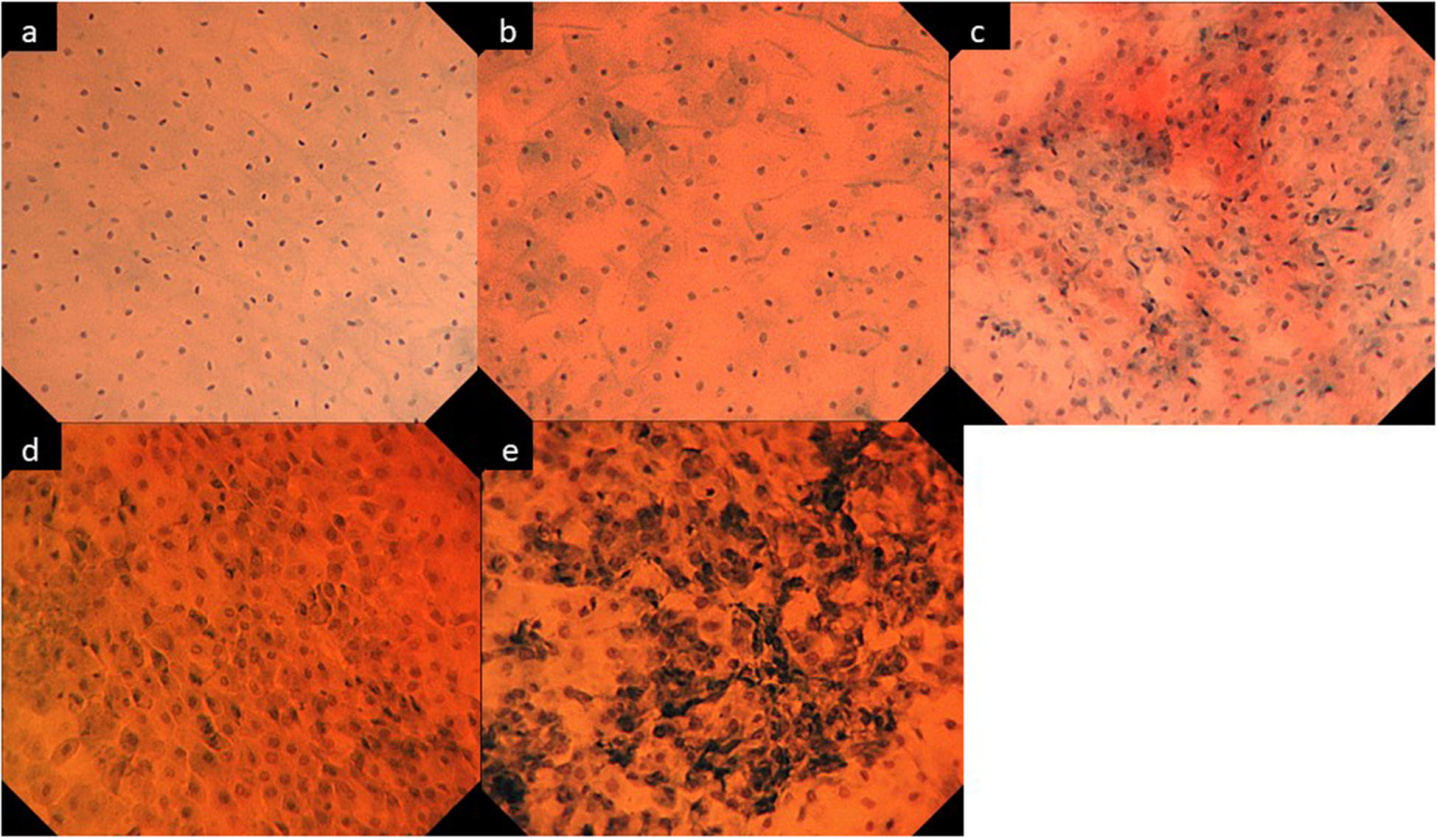
Classification of atypia by endocytoscopy. **a** ECA 1: Regular arrangement of small nuclei. **b** ECA 2: Different sizes of nuclei with halos. **c** ECA 3: Increase of cells without enlarged nuclei. **d** ECA 4: Slight increase of the ratio of nucleus ± cytoplasm. **e** ECA 5: Increase of the ratio of nucleus ± cytoplasm and irregular arrangement of enlarged and blurred nuclei

For evaluation of cell images, two images (from the most prominent site and surrounding area) per patient were selected, and the collection process was randomized and anonymous. Reviews were performed separately by three doctors (endoscopist: M. O., gynecologists: A. N. and pathologist: M. I.). Clinical information, preoperative results of cytology and histology, and other images including images obtained by colposcopy and endoscopy were unavailable at the time of the reviews. The three reviewers classified the cell images into 5 grades according to the ECA classification published by Inoue et al. [9]. ECA 4 and 5 are indicative of endoscopic malignancy. Figure 1 shows endocytoscopic images according to the ECA classification.

### Colposcopy

Colposcopy was performed by gynecologists before this study at an outpatient clinic using traditional equipment (OCS-500; Olympus, Tokyo, Japan). Punch biopsies were taken from abnormal lesions by using Atom Biopsy Punch, A-type (ATOM Medical Corporation, Japan).

### Histopathological diagnosis and final diagnosis

**Fig. 2:**
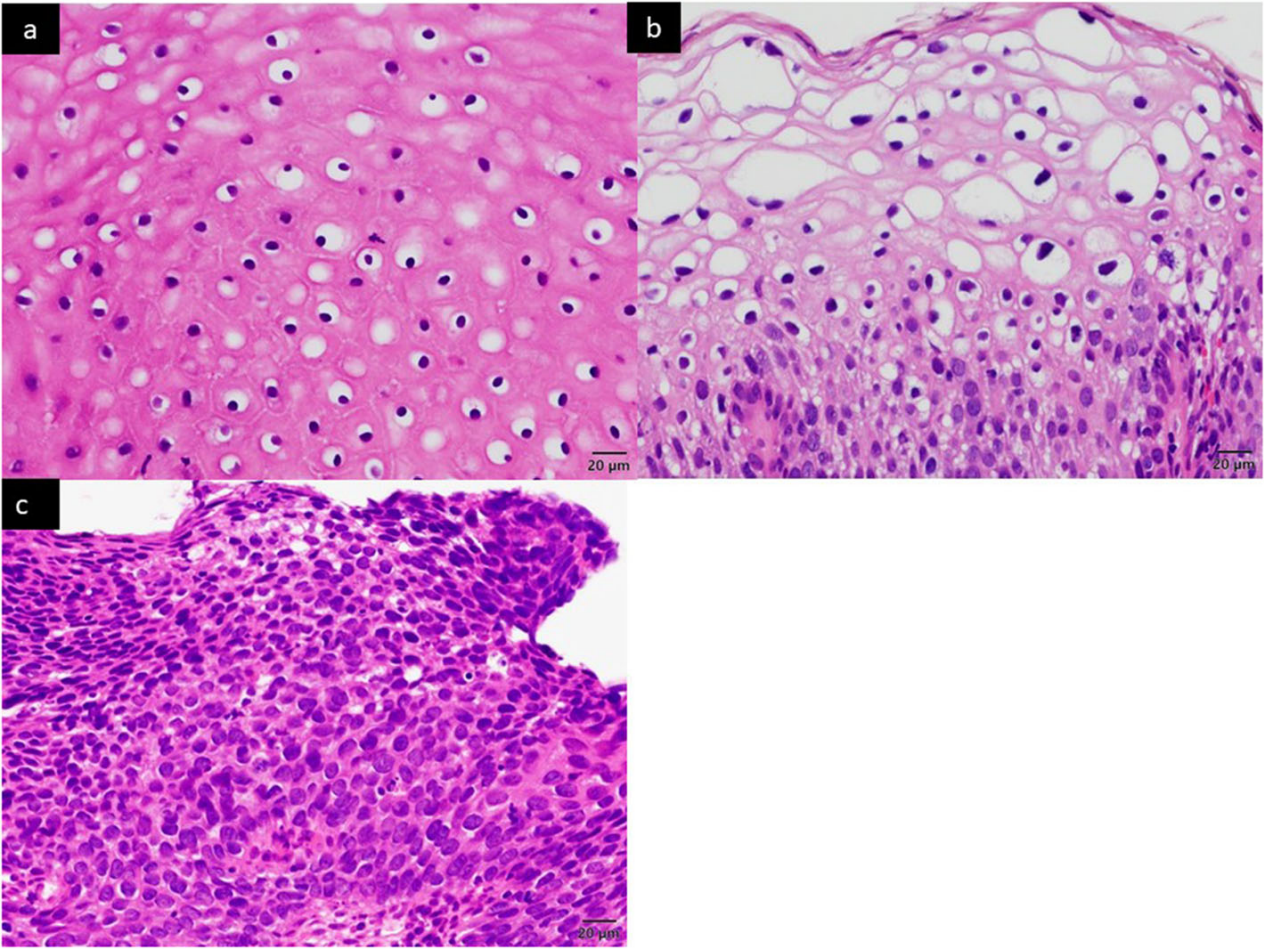
Histopathology of cervical intraepithelial neoplasia. Hematoxylin and eosin staining (× 400). **a** CIN 1. **b** CIN 2. **c** CIN 3

Histopathological diagnosis of biopsies and surgical materials was made according to the grades of CIN and VAIN by pathologists (Fig. 2).

### Outcomes

The primary endpoint was diagnostic accuracy (benign or malignant) of cell imaging at the most prominent site per patient judged by the reviewers to histological diagnosis using biopsies. When the results of the three reviewers did not match, the larger one of the reviews was used for calculation of accuracy. The secondary endpoints included diagnostic accuracy of all obtained cell images to histological diagnosis obtained by biopsies and colposcopy-guided biopsies, inter-observer agreement and adverse events. Malignant lesions were defined as ECA 4 and 5 in endocytoscopic images and as CIN 3 or VAIN 3 or worse in histology.

### Sample size

The sensitivity of colposcopy-guided target biopsy was about 70% according to a previous report [6]. With the assumption of diagnostic accuracy using cell imaging being 90%, the number of cases achieving a power of 80% at the significance level of 2.5% (one-sided) was calculated to be 34 based on results of the binomial test (normal approximation).

### Statistical analysis

JMP® Pro 11.2.0 (SAS Institute Inc.) was used for data analysis. For the primary endpoint, the Z test was performed at a significance level of 2.5% (one-sided) with respect to a threshold value of 70% (correct diagnosis rate by colposcopy-guided biopsy). Sensitivity, specificity, positive predictive value (PPV), negative predictive value (NPV) and accuracy were calculated, and the area under the curve (AUC) from receiver operating characteristic curve (ROC) analysis was evaluated. Fleiss’ *κ* was used to evaluate inter-observer agreement [14]. A *p* value of < .05 in each analysis was considered statistically significant.

## Results

### Patients

A total of 28 consecutive patients were enrolled to this study and 54 images excluding 2 images that were insufficient images were evaluated. The mean age of the patients was 43 (range, 25–68) years, and 2 patients showed local recurrences after the operation (cervical cancer and vaginal cancer). Colposcopy and biopsy were performed in 27 patients before enrolment, and CIN 3 or VAIN 3 or worse was diagnosed in 21 patients. Characteristics of the patients are shown in Table 1.

**Table 1:** Characteristics of patients

Numbers, n	28
Age (mean, range), years	43 (25–68)
Preoperative diagnosis, n	
VAIN	2
CIN	26
Diagnosis of colposcopy guided biopsy, n	
< VAIN 3 and < CIN 3	6
CIN 3	17
SCC	4
Intervention, n	
Cervical and resection of vagina	24
Mapping biopsy	4
Final diagnosis, n	
No dysplasia	4
CIN 2	1
VAIN 3 and CIN 3	19
SCC	4

VAIN: vaginal intraepithelial neoplasia, CIN: cervical intraepithelial neoplasia, SCC: squamous cell carcinoma

### Primary endpoint

Twenty-eight endoscopic images from the most prominent sites in the patients were analyzed. Table 2 shows the relationship between the ECS classification and grade of histological dysplasia. Sensitivity, specificity, PPV, NPV and accuracy of endocytoscopic images were 95.0% (84.8–98.6%), 87.5% (61.9–96.5%), 95.0% (84.8–98.6%), 87.5% (61.9–96.5%) and 92.9% (78.2–98.0%), respectively. Endocytoscopy significantly increased the diagnostic performance compared to colposcopy-guided biopsy (*P* < 0.01).

**Table 2:** Correlations between ECS classification and histological diagnosis (the most prominent sites)

Biopsy	< CIN 3 < VAIN 3, n	≥ CIN 3 ≥ VAIN 3, n	Total, n
ECS classification			
ECS 1–3, n	7	1	8
ECS 4, 5, n	1	19	20
Total, n	8	20	28

*CIN* cervical intraepithelial neoplasia, *VAIN* vaginal intraepithelial neoplasia, *ECS* endocytoscopy

Sensitivity; 95.0 (84.8–98.6), specificity; 87.5 (61.9–96.5), PPV; 95.0 (84.8–98.6), NPV; 87.5; (61.9–96.5), accuracy; 92.9 (78.2–98.0) (95% CI)

### Secondary endpoints

**Fig. 3:**
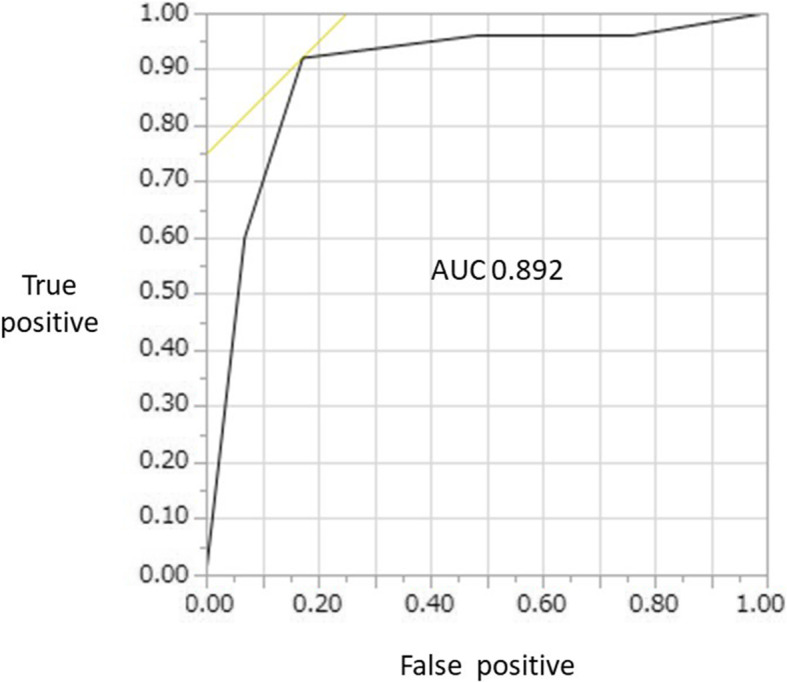
Receiver operating characteristic curve. ROC curve for diagnosing CIN 3 or VAIN 3 or worse in ECA 4 and 5. The area under the curve was 0.89

Excluding 2 images with poor staining, a total of 54 images including the most prominent site and surrounding area for each patient were evaluated (Table 3). Sensitivity, specificity, PPV, NPV and accuracy of endocytoscopic images were 92.0% (79.9–97.6%), 82.8% (72.4–87.5%), 82.1% (71.4–87.1%), 92.3% (80.7–97.6%) and 87.0% (75.9–92.2%), respectively. The ROC curve is shown in Fig. 3 and the AUC was 0.89.

**Table 3:** Correlations between ECS classification and histological diagnosis (including the most prominent sites and surrounding area)

Biopsy	< CIN 3 < VAIN 3, n	≥ CIN 3 ≥ VAIN 3, n	Total, n
ECS classification			
ECS 1–3, n	4	2	26
ECS 4, 5, n	5	23	28
Total, n	29	25	54

*CIN* cervical intraepithelial neoplasia, *VAIN* vaginal intraepithelial neoplasia, *ECS* endocytoscopy

Sensitivity; 92.0 (79.9–97.6), specificity; 82.8 (72.4–87.5), PPV; 82.1 (71.4–87.1), NPV; 92.3 (80.7–97.6), accuracy; 87.0 (75.9–92.2) (95% CI)

The relationship between colposcopy-guided biopsy and final histological diagnosis based on the operation is shown in Table 4. The accuracy of colposcopy-guided biopsy was 74.1% (64.0–84.0%), which was similar to that in previous reports. On the other hand, the accuracy of ECA classification to final diagnosis was 85.7% (73.2–85.7%).

**Table 4:** Correlations of histological diagnosis between colposcopy guided biopsy and final procedures

Final procedure	< CIN 3 < VAIN 3, n	≥ CIN 3 ≥ VAIN 3, n	Total, n
Colposcopy			
< CIN 3 < VAIN 3, n	2	5	7
≥ CIN 3 ≥ VAIN 3, n	2	18	20
Total, n	4	23	27

*CIN* cervical intraepithelial neoplasia, *VAIN* vaginal intraepithelial neoplasia

Sensitivity; 78.3 (72.3–84.1), specificity; 50.0 (16.0–83.5) PPV; 90.0 (83.2–96.7), NPV; 28.6 (9.1–47.7), accuracy; 74.1(64–84.0) (95% CI)

The diagnostic performance of each observer is shown in Table 5. Inter-observer agreement among the three reviewers using Fleiss’ *κ* was 0.78 (0.08–9.88, *P* < 0.01), and indicating good agreement. There were no adverse events and endocytoscopy had no adverse effects on scheduled procedures and final diagnosis.

**Table 5:** Diagnostic performance of each observer

Observer	Sensitivity	Specificity	PPV	NPV	Accuracy
A (endoscopist)	88.0 (75.4–95.1)	82.8 (71.9–88.9)	81.5 (69.8–88.1)	88.9 (77.2–99.5)	85.2 (73.5–91.8)
B (gynecologist)	92.0 (79.7–97.6)	79.3 (68.7–84.1)	79.3 (68.7–84.1)	92.0 (79.7–97.6)	85.2 (73.8–90.4)
C (pathologist)	95.7 (83.1–99.2)	77.4 (68.1–80.1)	75.9 (65.9–78.7)	96.9 (84.4–99.3)	85.2 (74.5–88.2)

*PPV* positive predictive value, *NPV* negative predictive value

## Discussion

This study demonstrated the potential of flexible endocytoscopy for digestive tract to evaluate CIN and VAIN. Endocytoscopy provides real-time cellular images in vivo and it enables immediate diagnosis and treatment without the need for biopsy. Endocytoscopy visualizes at a depth of about 50 μm from the surface, and atypical cells up to the surface such as CIN 3 and VAIN 3 can therefore be seen. Endocytoscopy might be useful for determination of the need for conization.

Recently, there have been a few reports on the usefulness of flexible magnifying endoscopy with narrow band imaging (ME-NBI) for evaluation of CIN [15, 16]. ME-NBI visualizes microstructures of the mucosa showing microvascular features with 80–100-fold. However, ME-NBI indirectly reflects histological findings and it is different from endocytoscopy to visualize cell atypia.

There have also been some reports on fluorescence confocal endomicroscopy of the cervix for evaluation of cellular images in vivo. J Tan et al. reported that the sensitivity for detection of CIN was 97%. However, low inter-observer agreement reported by Schlosser C et al. is problematic for a clinical setting [17, 18]. Endocytoscopy provides sharp images that reflect the size, chromatin concentration and density of the nucleus similar to histopathology, and it enables diagnosis of CIN in vivo. In addition, ECA classification was simple and useful for diagnosis of malignant lesions.

On the other hand, colposcopy, which has been one of the standard methods for diagnosis of CIN or VAIN for a long time, is a subjective and indirect test. We usually judge abnormalities with some typical findings (such as acetowhite epithelium, mosaic, atypical vessels) that indirectly represent cell concentrations and angiogenesis. The accuracy of colposcopy is therefore affected by the condition of observation and experience of the colposcopists.

In this study, colposcopy-guided biopsy was conducted by two well-trained gynecologist and showed accuracy that was similar to that in previous reports. The small number of colposcopists taking biopsies might have influenced the results.

Our study has some limitations. This study was performed by some experts at a single center with a small sample size, and colposcopy and endocytoscopy were not performed on the same day and the condition of the procedures were different. Generally, flexible endoscopies for digestive tract provides high-quality and high-resolution images, but the length of the scope is too long for gynecological use. Development of equipment suitable for diagnosis of gynecological neoplasia is needed. In addition, the diagnostic accuracies we provided were performed in selected patients and those were insufficient to compare each modality. Further study is needed to verify the usefulness of endocytoscopy for examination of and establishment of a treatment strategy for cervical and vaginal intraepithelial neoplasia.

## Conclusions

Optical cell diagnosis using flexible gastrointestinal endocytoscopy provides a high level of diagnostic accuracy, and ECA classification is clinically applicable. Endoscopic cellular imaging in vivo might be a new diagnostic tool for cervical or vaginal intraepithelial neoplasia.

## Supplementary information

**Additional file 2: Supplement figure.** Enrollment flowchart.

## Data Availability

The datasets used and/or analysed during the current study are available from the corresponding author on reasonable request.

## Abbreviations

CIN: cervical intraepithelial neoplasia
VAIN: vaginal intraepithelial neoplasia
PPV: positive predictive value
NPV: negative predictive value
ROC: receiver operating characteristic curve
AUC: area under the curve
ME-NBI: magnifying endoscopy with narrow band imaging
