# Supplementary figures and images for: In vivo optical cellular diagnosis for uterine cervical or vaginal intraepithelial neoplasia using flexible gastrointestinal endocytoscopy -a prospective pilot study-
Source: BMC Cancer. 2020 Oct 2;20:955. doi: 10.1186/s12885-020-07460-6 (PMC7530973; doi:10.1186/s12885-020-07460-6)

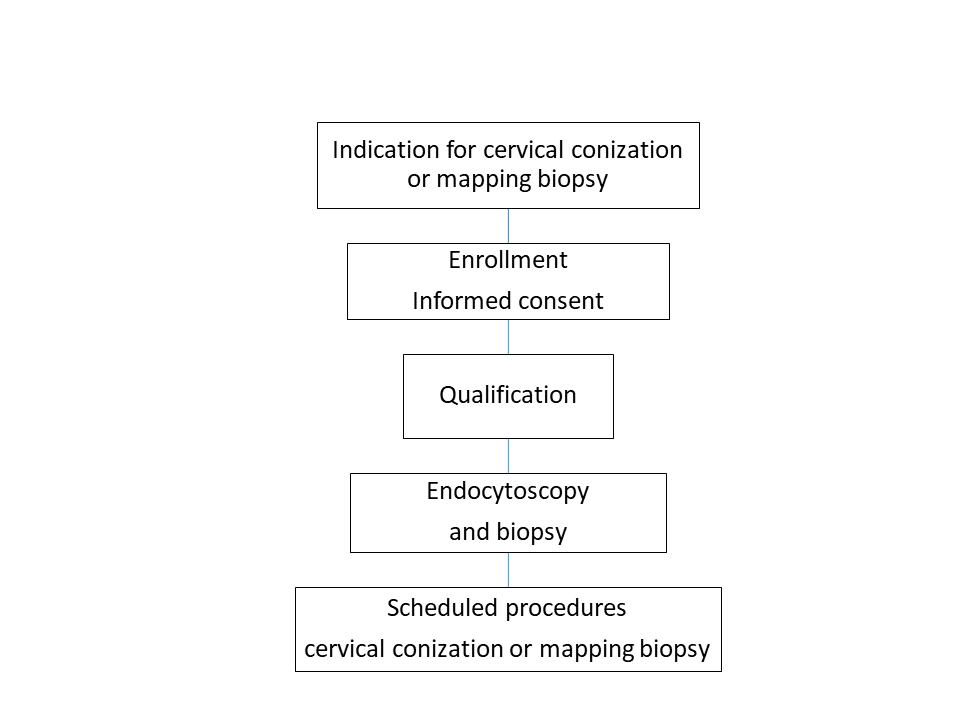

Supplement: Supplementary file 2 — Additional file 2: Supplement figure. Enrollment flowchart. [file 12885_2020_7460_MOESM2_ESM.tif]
